# Neurodevelopmental, Mental Health, and Parenting Issues in Preterm Infants

**DOI:** 10.3390/children10091565

**Published:** 2023-09-18

**Authors:** Richard J. Shaw, Soudabeh Givrad, Celeste Poe, Elizabeth C. Loi, Margaret K. Hoge, Melissa Scala

**Affiliations:** 1 Division of Child and Adolescent Psychiatry and Child Development, Stanford University School of Medicine, 401 Quarry Road, Stanford, CA 94305, USA; eloi@stanford.edu (E.C.L.); cpoe@stanford.edu (C.P.); 2 Division of Child and Adolescent Psychiatry, Weill Cornell Medicine, 525 E 68th Street, New York, NY 10065, USA; sog9022@med.cornell.edu; 3 Division of Neonatal-Perinatal Medicine, Department of Pediatrics, University of Texas Southwestern Medical Center, Dallas, TX 75390, USA; margaret.hoge@utsouthwestern.edu; 4 Division of Neonatal and Developmental Medicine, Stanford University School of Medicine, Palo Alto, CA 94304, USA; mscala@stanford.edu

**Keywords:** preterm infants, mental health, parenting, attachment, parent-infant interactions

## Abstract

The World Health Organization in its recommendations for the care of preterm infants has drawn attention to the need to address issues related to family involvement and support, including education, counseling, discharge preparation, and peer support. A failure to address these issues may translate into poor outcomes that extend across the lifespan. In this paper, we review the often far-reaching impact of preterm birth on the health and wellbeing of the parents and highlight the ways in which psychological stress may have a negative long-term impact on the parent-child interaction, attachment, and the styles of parenting. This paper addresses the following topics: (1) neurodevelopmental outcomes in preterm infants, including cognitive, sensory, and motor difficulties, (2) long-term mental health issues in premature infants that include elevated rates of anxiety and depressive disorders, autism, and somatization, which may affect social relationships and quality of life, (3) adverse mental health outcomes for parents that include elevated rates of depression, anxiety, and symptoms of post-traumatic stress, as well as increased rates of substance abuse, and relationship strain, (4) negative impacts on the parent-infant relationship, potentially mediated by maternal sensitivity, parent child-interactions, and attachment, and (5) impact on the parenting behaviors, including patterns of overprotective parenting, and development of Vulnerable Child Syndrome. Greater awareness of these issues has led to the development of programs in neonatal mental health and developmental care with some data suggesting benefits in terms of shorter lengths of stay and decreased health care costs.

## 1. Introduction

Global estimates of preterm (<37 weeks gestation) and low birth weight (LBW) infants range from 15–20% of all live births. Infants in this category have a two- to 10-fold higher risk of mortality than the term and normal birth weight infants and are at greater risk of medical complications and developmental problems including growth failure and developmental disabilities [1]. Preterm birth rates decreased between 2007–2014 but have increased since that date with one in 10 babies in the US being born prematurely. Rates of prematurity and low birth weight vary depending on race and ethnicity with higher rates in Black women [2].

While much attention has been focused on the medical and developmental issues of preterm infants, an appreciation of the psychological impact of the preterm birth and the neonatal intensive care unit (NICU) experience on the parents has been less well studied. This is reflected in the hospital NICU practices that have emphasized interventions to improve infant outcomes rather than the psychological health of the parents. However, the recent World Health Organization Recommendations for Care of the Preterm or Low-Birth-Weight Infant [3] have drawn attention to the need to address the issues related to family involvement and support including education, counseling, discharge preparation, and peer support.

The birth and hospitalization of a preterm or LBW infant in the NICU is typically an unexpected and traumatic experience for parents. Parents frequently report feelings of guilt, anxiety, and sadness about the loss of the “perfect” child. Sources of stress include aspects of the NICU environment, unexpected physical characteristics and behaviors of the infant, difficult interactions with NICU staff, and the inability to take on the expected parenting role [4]. The psychological models used to explain parental reactions include those of grief and loss, but also the trauma model, in which the baby’s preterm birth is experienced as a traumatic event.

In this paper, we review the often far-reaching impact of preterm birth on the health and wellbeing of the parents and highlight the ways in which psychological stress may have a negative long-term impact on parent-child interaction, attachment, and styles of parenting. A failure to recognize these issues may translate into poor outcomes that may extend across the lifespan.

## 2. Neurodevelopmental Outcomes

To provide a context to the impact of preterm birth, we start with a review of neurodevelopmental outcomes in the preterm infants. As improvements in survival have occurred among preterm infants, focus has shifted somewhat from preventing mortality to reducing neurodevelopmental impairment [5,6]. In the second half of gestation, brain volumes increase over 10-fold, making this a particularly vulnerable stage for neurological injury and disordered development [7,8]. The brains of preterm infants over time may show poor oligodendrocyte maturation, delayed myelination and neurite formation, and glial activation [9]. Rates of cognitive, motor, and sensory impairments are higher among preterm born than term born children and have been studied extensively [10,11,12]. The highest rates of impairment occur among the most premature, although even late preterm and early term born children may have outcomes below term norms. In a meta-analysis of studies performed after 2000, the rates of cognitive and motor delays were found in roughly 16% and 20% of preterm born children [10] with mild delays being more frequent than moderate to severe delays. Among those extremely preterm (EPT: born at less than 28 weeks gestation) and very preterm (VPT: born between 28–31 weeks gestation), the rates are higher and estimated to be 52% and 24%, respectively, in an international cohort [11].

It is important to note that challenges exist in interpreting the existing data regarding neurodevelopmental outcomes due to a variation in the individual center approaches to high-risk infants [13,14], varying definitions of impairment in the literature, changes in testing models, and a limited predictability of early-stage testing to predict school age outcomes [15,16,17]. There is a relative paucity of data on neurodevelopmental outcomes up to school age with some concern that early estimates of cognitive and motor impairment may underestimate issues identifiable at later ages [18,19]. In addition, the post discharge care environment may have a profound impact on the developmental trajectories, particularly for the highest risk infants [20,21,22].

Specific neurodevelopmental challenges among former preterm children include abnormalities of motor, cognitive, and sensory capabilities [6]. A composite of these three factors, often a combination of Bayley Scales of Infant Development (BSID) cognitive and motor scores and sensory impairment data, is commonly used as a primary outcome in neonatal research but misses other important challenges faced by preterm born children. Social, attentional, executive function, and communication skills may be undermeasured, either due to a test construct or the age of administration, but may represent more important functional and life-impacting issues to children and families. Attention has been brought by former NICU families on the need to include factors valued by the family, or those themselves who were born preterm, in the delineation of research outcomes [23,24,25].

Motor disturbances were among the earliest described and may encompass both cerebral palsy (CP), and movement disorders. The rates of CP have fallen in recent years with an estimate of 6.8% in preterm born children [10], down from previous estimates of ~11% [12] in more historical cohorts. Higher rates of CP are found in EPT-born children but have similarly fallen from estimates of ~14% [26] to 10% [10]. The rates of overall motor delay are higher than of CP, being up to 44% of EPT children and 16% of VPT children with moderate to severe delays in 11% and 6%, respectively [10]. Although movement disorders may be ameliorated by therapies, some may have a significant impact on daily living activities and are associated with an increased risk of reading and attention issues, speech and language impairment, and social-emotional problems [27,28].

Sensory impairments, including profound hearing and vision impairment, are less frequent than cognitive and motor delays but may have important long-term consequences for the preterm infant. Bilateral hearing impairment requiring intervention occurs in 1–9% of preterm infants [29]. As auditory input is important for language development, a failure to recognize and mitigate the deficits may have a significant impact on functional abilities and academic performance. Visual impairments, including acuity, convergence, and strabismus issues, are also more common in preterm infants and may lead to academic challenges [30,31].

Cognitive disabilities represent the most common neurodevelopmental impairment and may include difficulties in executive function, language processing, and working memory [18,32,33,34,35,36]. The rates of cognitive neurodevelopmental impairment are more common in EPT children with a pooled prevalence of 29% in recent studies, and 10.9% being moderate or severe. VPT children in this same review had a 14% rate of cognitive impairment with 5.8% being moderate or severe. Language skills are often commonly affected with VPT children who are eight times more likely to exhibit a poorer language trajectory during development [37]. IQ scores are 12–13 points lower than term infants in VPT born children [38] and up to 25 points lower for children born less than 26 weeks gestation [39].

Intelligence tests may fail to detect other issues that are important to cognitive function, including executive function, and a collection of abilities related to goal-direction and adaptive behavior. The included domains are working memory, impulse control, cognitive flexibility, planning, and organization. EPT children have higher rates of executive dysfunction at school age, particularly in working memory, planning, and organization [40]. Working memory, in particular, is a strong, independent predictor of academic achievement, even after accounting for IQ [41]. Attention disorders are more common among preterm infants and may significantly impact academic performance. VPT children have shown higher levels of attention problems, social impairment, and compromised communication skills than their term born counterparts [42].

## 3. Speech and Language Delays

Children born preterm demonstrate an increased risk for poor outcomes in language development. Early difficulties with language have been documented across all degrees of prematurity, including among children born extremely preterm [43,44], moderately and late preterm [45], late preterm [46,47], and preterm or at a low birthweight [48]. Children born preterm display difficulties on the measures of receptive language [43], expressive language [43,45,46], receptive and expressive language considered together [44,46], and articulation [43]. While an extensive literature documents the challenges that children born preterm experience with the acquisition of language skills, there is a paucity of research that has investigated how to mitigate the risk of prematurity on language development during the earliest opportunity to intervene—in the NICU environment [49]. More research is needed on NICU-based language interventions to provide children born preterm with a robust foundation from which to build language competence. 

## 4. Infant Mental Health Outcomes

Children born preterm demonstrate a heightened risk for a wide range of mental health and neurodevelopmental conditions. A variety of psychobiological factors have been implicated in the development and maintenance of these conditions. An appreciation of both the risk for and complexity of psychiatric and neurodevelopmental concerns can support the delivery of effective, tailored care to children born preterm.

### 4.1. Internalizing and Externalizing Disorders

Children born preterm may display behavioral difficulties as they progress through childhood, however, research findings in the literature are mixed. Several unique behavioral profiles have been detected. Some children born preterm may show the highest levels of behavioral problems in the period between two and three years of age [50]. However, Bosch and colleagues [51] found limited behavioral challenges among two-year-olds with a history of preterm birth. Some children born preterm may show few externalizing and internalizing behaviors, with relatively more internalizing behaviors as they age [52].

Variables that account for the outcomes in behavioral concerns include gestational age, skin-breaking procedures and morphine administration in the NICU [52], maternal depression, parenting stress, caregiver hostility, parental view of child vulnerability, and socioeconomic status [50,53]. The emerging research has focused on investigating the neural correlates of behavioral difficulties of the children born preterm. Results from Gilchrist and colleagues [54] have revealed that decreased neural network integration is linked to greater internalizing challenges when children are seven years of age.

Heightened risk for psychiatric disorders has been reported in the literature, although findings have been inconsistent. Among three- to six-year-olds, late preterm birth has been shown to relate to elevated odds of developing an anxiety disorder [55]. Late preterm birth has also been linked to elevated teacher reports of attention and internalizing concerns at six years [56]. Individuals born preterm display 1.5–2.9 times greater odds of developing depression, 2.7–7.4 times greater odds of developing bipolar disorder, and 1.6–2.5 times greater odds of developing psychosis [57]. The findings from Upadhyaya et al. [58] similarly demonstrate heightened risk for depression between the ages of 5 and 25 years in individuals born preterm. A history of preterm birth has also been found to relate to elevated odds of psychiatric hospitalization later in life [57]. Lower gestational age has been found to be linked to higher odds of psychiatric hospitalization [59]. However, in a report from Burnett and colleagues [60] on adolescents, mood and anxiety disorders were present at comparable levels between subjects who had been born preterm and subjects who served as controls.

### 4.2. Social Relationships and Autism

The extant literature has demonstrated a link between a history of preterm birth and social functioning. At seven to nine months of age, children born preterm demonstrate lowered social attentional preference relative to children born full term, although this difference is no longer present at five years [61]. Preterm birth is also related to an elevated risk for autism spectrum disorder (ASD; [62]). One report indicates a prevalence rate of ASD of 28–40% in a local sample of adolescents with a history of preterm birth [63]. Research from Chang and colleagues [64] has demonstrated that earlier gestational age is linked with an elevated risk for ASD. Among the children with ASD, the children born preterm show poorer nonverbal behaviors but better socioemotional reciprocity and peer relationships relative to the children born full term [62]. The candidate etiological pathways for the onset of ASD in children born preterm are underlying inflammatory and genetic processes [65,66].

### 4.3. Somatization

Given that children born preterm undergo a multitude of painful procedures over the course of a NICU admission, it is critical to understand the degree to which early, concentrated experiences of pain are related to later pain processing and management. Grunau and colleagues have found that young children with a history of extremely low birth weight and preterm birth demonstrate clinically elevated levels of somatization [67]. Variables that have been found to relate to somatization in the children born preterm include family relations, maternal sensitivity, and NICU admission history [67].

### 4.4. Quality of Life

Quality of life is an important consideration when caring for infants born preterm who have a greater likelihood of enduring the painful and extensive medical interventions from the earliest moments of life. An examination of the quality of life of individuals born preterm has resulted in mixed findings, depending in part on the source of data and the age of the subjects. The parents of children born preterm have indicated a lower quality of life for their children at 10 years of age as compared to the parents of children born full-term [68]. Subject- and caregiver assessment of quality of life has demonstrated a less favorable quality of life relative to the controls at 13 and 26 years [69]. On the other hand, Roberts and colleagues [70] reported that adolescents with a history of extremely preterm or extremely low birth weight have quality of life ratings that are comparable to control peers. Similarly, adults with a history of moderately preterm birth demonstrate a quality of life that is similar to their peers with a history of full-term birth [71].

The early life experiences of children born preterm, and their families may contribute to cascading consequences in the areas of mental health and neurodevelopment. It is important to recognize that the etiology of psychiatric and neurodevelopmental conditions is often multifactorial, encompassing factors across the social and biological realms. A keen understanding of the key factors and processes that contribute to the differences in development can facilitate the development of interventions that ameliorate the impact of preterm birth and promote child and family well-being. Parental and child health are inextricably linked. In order to support the development of children, clinical attention should also address the needs of parents.

## 5. Parental Mental Health Outcomes

When considering mental health issues related to preterm birth, it is important to take into account the impact on parents. For many parents, an infant’s admission to the NICU can evoke feelings of shock, guilt, fear, sadness, and helplessness. In summarizing the NICU parent experience, Miles [72] categorized stressors for NICU parents into four categories: (1) the infant’s appearance and behaviors, (2) the sights and sounds in the NICU, (3) parental relationship and communication with staff, and (4) parental role alteration. NICU parents are faced with seeing their sick infant exposed to intensive medical intervention in an unfamiliar environment, while simultaneously learning how to effectively communicate with staff, and to trust in one’s own abilities as a parent. If unaddressed, the mental health sequelae of these stressors can disrupt a parent’s ability to be present and engaged in their infant’s care, potentially causing a negative impact on both the short-term and long-term child-parent relationship, child developmental outcomes, and overall parent mental health [73,74,75].

### 5.1. Grief and Loss

Experiences of grief and loss are also commonly reported by NICU parents. For NICU parents, the time around the end of their infant’s life can be especially challenging due to issues related to decision making, saying goodbye to their infant, and making preparations for after the death [76,77]. In addition, NICU parents have reported experiencing anticipatory grief, or the psychological challenges associated with hoping for the infant’s survival while simultaneously preparing for their death [78]. Ambiguous loss is also commonly reported by NICU parents, including feelings of loss related to important milestones or experiences such as the imagined pregnancy or birth, having a baby shower, or being able to hold one’s baby immediately after birth [79,80]. Additionally, the developmental trajectories of infants in the NICU related to prematurity or other complex medical needs can often be very different than what a parent imagined for their child, themselves, and their family [81].

### 5.2. Depression

Despite what can often be a very vulnerable time for all new parents, stressors unique to the NICU experience likely contribute to the higher reported rates of depression among NICU parents when compared to the general population [82]. For example, when compared to the parents of full-term infants, the parents of very premature infants reported much higher rates of depression shortly after birth [83]. Moreover, while approximately one in seven mothers and one in 10 fathers in the general population experience postpartum depression [75], this number may be as high as four in 10 mothers of preterm infants [84]. With reported feelings of inadequacy, helplessness, and guilt, depressive symptoms have been found in as many as 38% of all NICU parents [85], often with depressive symptoms decreasing over time [86,87].

### 5.3. Anxiety and Traumatic Stress

In addition to symptoms of depression, parental stressors associated with an infant’s NICU admission have been reported to lead to increased rates of parental anxiety and traumatic stress. Malouf and colleagues [88] found that among NICU parents, 41.9% reported experiencing anxiety, and 39.9% experienced post-traumatic stress. Critical medical diagnoses such as prematurity, traumatic birth experiences, and witnessing infants receive intensive medical intervention can lead to higher rates of anxiety and traumatic stress that may meet criteria for acute stress disorder or post-traumatic stress disorder (PTSD; [89,90,91,92]). Commonly reported traumatic stress symptoms include symptoms of arousal and intrusion, as well as either a difficulty leaving the infant’s bedside or an avoidance of the NICU [93,94]. Despite remaining higher than the general population of parents, NICU parent reports of anxiety and traumatic stress have also been found to decrease over time [88]. Lefkowitz and colleagues [95] found that while 35% of mothers and 24% of fathers met the criteria for acute stress disorder a few days after their infant’s NICU admission, when screened 30 days later, 15% of mothers and 8% of fathers went on to meet the criteria for PTSD.

### 5.4. Substance Use

In the United States, every 25 min a baby is born who will experience symptoms of Neonatal Abstinence Syndrome (NAS) due to the discontinuance of in-utero exposure to substances [96,97]. Parents of these children are often forced to find ways to cope with their infant’s prolonged NICU admission, as well as with managing their own psychological adjustment and substance use [98]. While little is known about the link between traumatic stress and substance use in NICU parents specifically, the literature suggests that the prevalence of PTSD among those with substance use disorders can range from 25.3% to 49% [99]. In addition to potentially suffering from the biopsychosocial consequences of addiction [100], a newborn’s withdrawal symptoms and need for intensive and sensitive care can cause a parent distress, guilt, and create challenges for a parent’s ability to bond and connect with their baby [101,102].

### 5.5. Relationship Strain

Parenting a child with a serious or chronic illness increases the risk for breakups or divorce [74,103]. The relationship strain experienced by NICU parents can be especially challenging because the infant has never left the hospital and parents have yet to experience their baby on their own and may be excluded from care [104]. The differences in coping styles, gender roles and expectations, and communication styles can add additional stress for parents [105]. For example, some fathers are forced to return to work while also feeling responsible for caring for the mother, the newborn baby, and older children [106]. In addition, many NICU hospitalizations can last for months at a time, placing increased strain on the parents to make arrangements for other children and to navigate a return to work, potentially causing parents to be separated from each other for long periods of time [107]. The social and emotional strain placed on NICU parents can persist after discharge and have lasting effects on family relations, including the critical parent-infant relationship and attachment.

## 6. Parent-Infant Relationship and Attachment

The quality of caregiving relationships during infancy and early childhood has significant and lasting psychological and biological impacts on the developing child. The parent-infant relationship is one of the infant’s most proximal environmental exposures, and preterm infants are considered to be neurologically and biologically more vulnerable to their environmental exposures, hence [108], it is critical to understand the barriers and challenges in developing optimal relationships in the NICU parents and infants. Prematurity, particularly when leading to a NICU admission, can cause a disruption in the normal process of parent-infant bonding [109]. Preterm infants are less interactive, less alert and, and more easily dysregulated, and as a result their parents can have a harder time reading their cues [110,111]. NICU admission leads to parent-infant separation and makes it challenging, and at times impossible, for parents to hold their infant and to help soothe or regulate them when in distress [112]. The parents of preterm infants also experience higher rates of psychiatric distress [86] and can experience lower parental self-efficacy (parent’s self confidence in being able to carry out their parental role) [113] which can add to their difficulties in bonding with their infant.

The parent-infant relationship is complex and multidimensional. Maternal sensitivity (defined as the mother’s ability to detect, interpret, and respond to their infant’s emotional and physical needs [114]), the quality of the parent-infant interactions, and the patterns of attachment are among the main dimensions studied in both the general and the preterm parent-infant population. Sensitive parenting and high-quality parent-infant interactions are associated with better neurocognitive, socioemotional, and language development, and higher academic achievement later in childhood in preterm infants [115,116,117,118]. Inversely, less sensitive parenting has been associated with an increase in externalizing behaviors in early childhood, and in particular, in those preterm infants who experience higher levels of distress in infancy [119]. Drawing on this literature and our general understanding of the effects of the parent-infant relationship in full-term infants, higher levels of parental sensitivity and higher quality of parent-infant interactions are thought to be protective factors in the face of the increased developmental risks that preterm infants face.

Given the importance of high-quality parent-infant relationships in the NICU population and the many challenges these infants and parents face in establishing an optimal bond in the beginning, many researchers have looked at the various aspects of the parent-infant relationship in this population to discern if there are any differences when compared to the general population. The results are heterogenous and difficult to interpret. The heterogeneity in the results is likely due to several factors: (1) as mentioned above, the parent-infant relationship is complex and multidimensional and therefore, different studies have looked at different aspects of this relationship, and even those that have looked at the same dimension, at times, have used different assessment tools and methods, (2) among preterm infants there is a significant diversity in terms of the degree of prematurity, medical comorbidities, and the length of stay in the NICU, all of which can affect the quality of the parent-infant relationship, (3) NICUs and the supportive/therapeutic services they offer (family based developmental care practices, mental health services, psychosocial support services, etc.) differ widely, (4) factors such as race, ethnicity, and psychosocial adversity play an important role in the quality of the care patients receive and in their outcomes, and (5) differences in the study designs in terms of the timeline of assessments, and whether the study is longitudinal vs. cross sectional, and if longitudinal the follow up timelines can all create a heterogeneity in the findings. Here, we summarize some of the findings on the three core dimensions of the preterm parent-infant relationship.

### 6.1. Preterm Parent-Child Interactions

The majority of the studies that look at the preterm infant behavior have found preterm infants to be less interactive, less responsive, and to demonstrate less positive affect [120,121]. However, some studies found no differences between the preterm and full-term infant behaviors and a small number of studies found mixed results, or more favorable infant behavior among the preterm infants [122,123,124]. It is important to note that the degree of prematurity, other medical comorbidities, pain and distress, or sedation can all impact the degree of a preterm infant’s responsiveness and engagement in dyadic interactions. A larger number of studies have looked at the maternal interactive patterns in the mothers of preterm infants. The findings here are more heterogeneous and therefore, it is not easy to draw any universal conclusions based on these studies. About half the studies have shown less favorable maternal interactive patterns such as lower sensitivity, more controlling and intrusive behavior, and lower responsiveness [125,126]. There are, however, studies that show higher levels of attunement and maternal sensitivity, and responsiveness in the mothers of preterm infants, and a fair number of studies that have found no statistically significant differences between these mothers and the mothers of full-term infants [125,127,128,129,130,131]. Finally, a smaller subset of studies has looked at the quality of the dyadic interaction in the preterm population. About half of these studies have found a lower quality of dyadic interactions in these mothers and infants [121,122,123,124,125,126,127,128,129,130,131,132,133]. These studies have found less dyadic coregulation, less cooperation, synchrony, and positive affect in the preterm mothers and infants. Others have found no statistically significant differences, although the majority of the studies that found no differences were performed when infants were six months or older [134,135,136].

Looking at the findings of the research on preterm parent-infant interaction highlights the fact that preterm mothers and infants constitute a heterogeneous population. There are differences in the infants’ medical condition and birth weight, parents bring their own varying psychosocial and personal backgrounds and histories of trauma or adversity, and the NICUs differ significantly in terms of the resources (including early screening, psychological support, and interventions) that they provide. The timing of assessment can significantly affect the findings: while preterm infants are less interactive and neurologically premature, in many cases they eventually catch up with their full-term counterparts. Similarly, during the early postpartum period and the NICU admission, many parents experience higher degrees of psychological distress and uncertainty about their infant’s developmental and medical outcomes. Therefore, depending on the population studied and considering the variations in methodological designs discussed earlier, it is not surprising to see the heterogeneity in the findings.

Nevertheless, a number of points can be more definitely concluded based on these studies: (1) preterm infants, in particular very preterm and extremely preterm infants and those with medical complications, contribute substantially less to the dyadic interactive flow and use different ways of communicating their needs and distress. This in turn, can affect parental interactive patterns with these infants, (2) there are subsets of vulnerable groups among parents of the preterm infants when it comes to parental sensitivity and interactive style. Some of the factors leading to vulnerability are better known, however, we need to better understand which parental and infant factors can lead to an increased vulnerability in developing optimal parent-infant interactions in the preterm population, and (3) preterm infants and their parents may undergo periods during which the quality of their interactions are more challenged (including during the NICU stay, the immediate period post-discharge, and the times when there are medical crises or complications). These periods of increased vulnerability need to be better studied and understood.

### 6.2. Preterm Parent-Infant Attachment

Another important framework to assess the parent-infant relationship is through attachment classification. Attachment theory and science describe the role that parents play for their infants in making them feel safe, secure, and protected [137]. Children who consistently receive sensitive, loving, and responsive parenting are able to use their parents as a safe haven when feeling in danger and a secure base from which to explore their environment. These children develop what is classified as a secure attachment. Unlike children who develop secure attachment, those who develop insecure attachment often are faced with inconsistent or distant, insensitive, or unresponsive caregiving. Broadly, the insecurely attached children are divided into the anxious-ambivalent group (children who have received an inconsistent quality of responsiveness and sensitivity and therefore act in ambivalent ways toward their caregivers) and the anxious-avoidant group (children who have received an insensitive, unresponsive, and absent caregiving who are unable to use their caregiver as a safe haven or a secure base). A fourth category of disorganized attachment was later added to this classification. Children who have disorganized attachment style often have caregivers who are at times frightening or frightened due to their own significant history of unresolved trauma. These children do not have an identifiable pattern of relating to their caregivers at times of separation, reunion, or distress. Even though a disorganized attachment is the only category that is directly associated with later psychopathology, insecure attachment styles are also associated with problematic patterns of emotional regulation, interpersonal, and academic skills. The gold standard for the assessment of attachment style is the Strange Situation Protocol (SSP) which is often used when the infant is nine to 18 months old [113].

Many of the studies that have looked at the preterm infant’s attachment styles have reported higher rates of insecure attachment in this group compared to the full-term infants [138,139,140,141,142]. Studies have also found higher rates of disorganized attachment [143,144]. However, these findings are not consistent, and some research has not demonstrated any statistically significant difference in the rates of the various categories of attachment styles between the preterm and full-term infants and their caregivers [144,145,146]. Looking more closely at some studies, there are again subpopulations of preterm infants who might be at a higher risk of developing insecure or disorganized attachment styles: VLBW infants, infants with respiratory illness, those with longer lengths of hospitalization, and children with more significant developmental delays [147,148]. These findings highlight the importance of understanding the infant, parental, and environmental factors that can impede or promote the child’s attachment to their caregiver. Identifying the infants and parents who are biologically, medically, developmentally, or psychologically at risk of developing insecure or disorganized attachment styles can help us tailor interventions and support systems specific to their needs.

## 7. Impact on Parenting

In addition to the impact of preterm birth on the attachment and parent-child interactions, it is important to consider how these early life experiences for both parent and child affect parenting behaviors. Parents have a critical impact on an infant’s learning and development through parenting interactions. Parental emotional trauma during a neonatal intensive care unit (NICU) admission often has a significant impact on the parents’ mental health and distorted parental perceptions of their child’s vulnerability (PPCV). This impacts their parenting styles and can result in a style of overprotective parenting. NICU parents are at a high risk for developing increased PPCV. Parents of preterm infants had significantly higher PPCV for their healthy children at age 36–42 months old compared to healthy term infants [149]. Sixty four percent of the mothers of ex-premature infants viewed their children as vulnerable in one study [150]. Additionally, about 83% of mothers who experienced significant emotional trauma during the NICU stay also say they have distorted vulnerability views of their infants [151,152]. It has been found that the medical complexity of the infants does not correspond with the PPCV ratings, and that NICU parents have high ratings of PPCV compared to healthy term infants [153].

The effects of increased PPCV on the parents and child can persist after the infant’s discharge from the NICU, such as compromises in optimal parenting skills and stunted learning and developmental outcomes for the child. This is described in the concept of Vulnerable Child Syndrome (VCS). Green et al. [154] first described the theory that VCS affected parents with children whose ages and diagnoses varied, but that a fear for the child’s survival persisted even after the resolution of a traumatic health event. This fear led to increased PPCV and then overprotective parenting skills. The final common point was associated poor outcomes for the child’s behavior, social skills, over somatization of bodily symptoms, school problems, health care utilization, and psychological problems. In 2015, Horwitz et al. [155] developed a theoretical model specifically for VCS in NICU children and showed that the maternal responses and sequelae to traumatic events, maternal dysfunctional coping methods to trauma, and the levels of family support were most influential in the development of VCS, per a multi-regression analysis model. Hoge et al. [156] have further explored the concept of utilizing trauma-informed cognitive behavioral therapy models to predict the risk and progression of the development of VCS.

The reported incidence of VCS in the general pediatrics literature has been around 10–21%; however, the incidence in the NICU families is unknown [157]. Given that the risk factors of anxiety, depression, trauma, and distorted views of vulnerability are high in this population, more so than the general population, it could be assumed that the incidence is at least as high as in the general population, and likely higher. Thus, it is important to support the NICU parents during the infant’s hospital stay by finding ways to ameliorate their ability to effectively cope with the emotional trauma during, and after a NICU admission, and help them have realistic and healthy perceptions of their child for the future.

Cognitive behavioral therapy (CBT) could be an effective mode of treatment to prevent VCS in the NICU population. Manualized CBT has been shown to be feasible to address concepts of PPCV and VCS in the NICU parents of premature infants with very high parental satisfaction [158]. These parents have expressed stories of utilizing the techniques and improving situations once discharged from the NICU. Ongoing analysis is underway to assess the effects on PPCV scores and long-term outcomes of the children.

## 8. Discussion

With increased rates of survival of preterm infants, attention is now being focused on the long-term issues affecting both infants and their caregivers. These include not only chronic medical complications and neurodevelopmental delays, but also the parenting and mental health issues that have been referenced above. For many parents, the trauma of a preterm birth may have a lifelong impact, not only on styles of attachment and parenting approaches, but also on their own mental health and well-being.

Interest in these issues has led to the growth of new specialties, including neonatal mental health and developmental care. While still a relatively young field, it is fortunate that researchers are starting to develop a number of effective and evidence-based interventions that have the potential to improve both the infant and parent outcomes. These include: (1) developmental care interventions involving measures to reduce infant pain and stress, sensory interventions to stimulate development, and educational interventions that teach parents how to recognize their infant’s developmental needs and foster healthy parenting skills [156], (2) interventions that include parent-infant psychotherapy that address the relationship and interactions between the parent and infant with the goal of fostering parental sensitivity and engagement, and (3) interventions directed specifically at the parents to address parental stress and trauma.

Although these interventions have proven efficacy and long-term benefits, access to psychological and developmental care services is not uniform across the NICUs. Even in those hospitals that fund psychological services, there are often gaps and disparities in their implementation and utilization based on cultural and systemic variables. In part, these gaps exist due to the absence of robust mental health screening for parents. Although many obstetrical programs now offer screening for depression, it is rare for the NICUs to routinely screen parents of preterm infants, in particular, the non-birth parents who may be equally impacted by the birth trauma. In addition, access to follow-up mental health care after the infants are discharged is often variable and, in many cases, completely absent. Similarly, preventative mental health care in the prenatal period is generally not available even in well-funded academic programs.

Looking forward, there is a strong need for research and program development in the areas of neonatal mental health and developmental care. Although there is some data that has shown shorter lengths of stay and decreased health care costs, there has been no systematic evaluation of the risks associated with not offering early intervention or the potential benefits of providing these services. Patterns of overprotective parenting and symptoms of VCS, for example, as described above, have been linked with the overutilization of healthcare services in pediatric care, as well as increased rates of somatization, which also burden the healthcare system. However, without robust evidence to demonstrate the financial benefits of early childhood and parent interventions, it will be difficult to convince both hospital programs and insurance companies to provide adequate mental health care and parent support. Future research would do well to demonstrate the benefits of mental health and developmental care interventions for the well-being of infants, families, and the health care systems that serve them.

## Data Availability

Not applicable.
